# Wild relatives to improve heat tolerance of cultivated quinoa (*Chenopodium quinoa*): pollen viability and grain number

**DOI:** 10.1093/jxb/eraf235

**Published:** 2025-06-02

**Authors:** Jiemeng Xu, Hafiz Umar Farooq, Mariam Hashim, Elodie Rey, Ramiro Curti, Angel Morris, Peter J Maughan, Eric N Jellen, David E Jarvis, Daniel Bertero, Vanessa Melino, Mark Tester

**Affiliations:** Center for Desert Agriculture, King Abdullah University of Science and Technology, Thuwal 23955, Saudi Arabia; Global Wheat Program, International Maize and Wheat Improvement Centre (CIMMYT), Texcoco 56237, Mexico; Center for Desert Agriculture, King Abdullah University of Science and Technology, Thuwal 23955, Saudi Arabia; Center for Desert Agriculture, King Abdullah University of Science and Technology, Thuwal 23955, Saudi Arabia; Center for Desert Agriculture, King Abdullah University of Science and Technology, Thuwal 23955, Saudi Arabia; National University of Salta, Av. Bolivia 5150, A4400 Salta, Argentina; Center for Desert Agriculture, King Abdullah University of Science and Technology, Thuwal 23955, Saudi Arabia; College of Life Science, Brigham Young University, Provo, UT 84602, USA; College of Life Science, Brigham Young University, Provo, UT 84602, USA; College of Life Science, Brigham Young University, Provo, UT 84602, USA; Department of Plant Production, Faculty of Agronomy, University of Buenos Aires, C1053 Cdad. Autónoma de Buenos Aires, Argentina; Center for Desert Agriculture, King Abdullah University of Science and Technology, Thuwal 23955, Saudi Arabia; School of Environmental and Life Sciences, College of Engineering, Science and Environment, University of Newcastle, Callaghan, NSW 2308, Australia; Center for Desert Agriculture, King Abdullah University of Science and Technology, Thuwal 23955, Saudi Arabia; INTA-CONICET, Argentina

**Keywords:** *Chenopodium berlandieri*, *C. hircinum*, *C. quinoa*, grain number, heat tolerance, high temperature stress, pollen viability, seed set, yield

## Abstract

Quinoa (*Chenopodium quinoa*) is well-known for high nutritional value and wide adaptability, but it is considered to be heat sensitive. To address this issue, accessions from two tetraploid wild relatives, *C. berlandieri* and *C. hircinum*, both native to hot environments, were evaluated alongside lowland and highland ecotypes of cultivated quinoa under field conditions with differing planting dates. *Chenopodium berlandieri* showed the best yield under the extreme heat experienced during the last planting, followed by lowland quinoa, *C. hircinum*, and highland quinoa. The yield advantage of *C. berlandieri* was achieved by maintaining higher grain number/seed set. Pollen viability was positively correlated with seed set under heat stress in cultivated quinoa, indicating its limiting effects. Considerable variation was observed for pollen viability among representative accessions of each species/ecotypes after a 38/33 °C day/night treatment for 5 d, ranging from an 80% reduction observed in highland quinoa to a 30% reduction in *C. berlandieri*. The most heat-sensitive period for pollen viability was 8–10 d before flowering, corresponding to the early pollen mother cell stage and it was conserved among the different species. *In vitro* pollen germination tests also demonstrated the heat tolerance of *C. berlandieri*. Taken together, our results suggest that wild relatives, particularly *C. berlandieri*, could be crossed with cultivated quinoa to introduce reproductive heat tolerance.

## Teaser

Wild relatives of quinoa maintain pollen viability, seed number, and grain yield under heat stress, with pollen viability potentially being a key trait to improve heat tolerance of cultivated quinoa.

## Introduction

Global surface temperatures have been rising rapidly over recent decades, with the highest average being recorded in 2023 (NOAA National Centers for Environmental Information, 2024). This has been accompanied by more intense and frequent heat waves (IPCC, 2023), and consequently plants are being increasingly exposed to temperatures in excess of physiological thresholds, causing heat stress (Wahid *et al.*, 2007). Heat stress imposes detrimental effects on plant growth and development, especially on reproductive development, which can directly translate to yield loss in crops (Satake and Yoshida, 1978; Begcy *et al.*, 2019; Xu *et al.*, 2022). It has been shown that crops are likely to be significantly affected by heat unless appropriate mitigation measures are taken (Coughlan de Perez *et al.*, 2023; Luo *et al.*, 2023). This necessitates effective adaptation strategies to improve resilience under hot temperature conditions, such as the selection and breeding of heat-tolerant crops.

Quinoa (*Chenopodium quinoa* Willd., 2*n*=4*x*=36, AABB genome) is a semi-domesticated pseudo-cereal that originated in the Andes and is known as the ‘mother grain’ by indigenous Andean peoples. Due to its high nutritional value and adaptation to harsh high-altitude environments (such as drought, frost, and salinity), its cultivation has expanded to many other countries (Bazile *et al.*, 2016; Alandia *et al.*, 2020), and it has gained increased interest from the research community (Jarvis *et al.*, 2017; Patiranage *et al.*, 2022; Rey *et al.*, 2023). However, quinoa cultivation is still restricted to cool areas due to its heat sensitivity compared with other major crops such as wheat and rice. High temperature treatments, imposed either by late sowing in field experiments or by temperature control within growth chambers, have shown negative effects on yield production of quinoa (Lesjak and Calderini, 2017; Hinojosa *et al.*, 2019; Alvar-Beltrán *et al.*, 2020; Tovar *et al.*, 2020; Matías *et al.*, 2021; Abbas *et al.*, 2024). Heat-induced yield losses are often associated with reduced grain size and, less frequently, with reduced grain number and underlying pollen defects (Lesjak and Calderini, 2017; Tovar *et al.*, 2020). There are contrasting reports on the effects of heat on pollen viability, primarily due to a lack of examination of stage-specific heat sensitivity during pollen development (Hinojosa *et al.*, 2019; Tovar *et al.*, 2020). Studies of other crops have indicated that pollen viability is the most important factor determining seed number, and hence yield, under reproductive heat-stress conditions (Jagadish *et al.*, 2007; Xu *et al.*, 2017, 2022; Begcy *et al.*, 2019), and thus it is worthwhile determining whether the same applies to quinoa.

Wild relatives are often considered to have greater genetic diversity when compared with domesticated crops, making them valuable sources of beneficial traits and alleles for crop improvement. For example, exotic alleles from *Aegilops tauschii* have increased yield and reduced canopy temperature of common wheat under heat (Molero *et al.*, 2023), and the early-morning flowering trait from wild *Oryza officinalis* has helped to alleviate heat-induced sterility (Ishimaru *et al*., 2010). In addition, *THERMO-TOLERANCE 1* (*TT1*) from *O. glaberrima* enhances heat tolerance through more efficient removal of denatured cytotoxic proteins (Li *et al.*, 2015).

It has been hypothesized that an ancestral AABB tetraploid *C. berlandieri* (pitseed goosefoot) experienced long-distance dispersal to South America, where it diverged into *C. hircinum* (avian goosefoot), and was eventually domesticated as quinoa (Maughan *et al*., 2024). This domestication could have occurred either through a single event that gave rise to highland and lowland quinoa sequentially, or through two independent events that led to the formation of the two ecotypes separately (Jarvis *et al.*, 2017). Quinoa is still considered as a partially domesticated crop and possesses large genetic diversity due to its ability to hybridize with its wild ancestors *C. berlandieri* and *C. hircinum*, which are widely spread across temperate and subtropical North and South America (FAO & CIRAD, 2013; Young *et al.*, 2023; Maughan *et al.*, 2024). These wild relatives show very diverse phenotypes and include ecotypes that occupy very warm environments such as the Sonoran Desert, the Gulf of Mexico coast, and the Gran Chaco (Curti *et al.*, 2022; Maughan *et al.*, 2024). Many of these strains have now been reposited in gene banks, such that genomic resources of quinoa and its wild relatives are becoming increasingly abundant (Jarvis *et al.*, 2017; Rey *et al.*, 2023; Young *et al.*, 2023). Maughan *et al*. (2024) found that quinoa passed through a pronounced genetic bottleneck during domestication. Hence, we hypothesize that they probably harbor previously unexplored mechanisms of adaptation to high temperatures that are not present in, but could potentially be transferred into, cultivated quinoa.

To select quinoa and its wild relatives with elevated heat tolerance and identify associated physiological traits, a panel of 16 accessions was evaluated for yield performance and its association with grain number when exposed to heat treatments in the field in Saudi Arabia. The relationship between grain number/seed set and pollen viability was then determined in cultivated quinoa, followed by identification of the genetic variation of pollen viability among five representative accessions under stage-specific heat treatments. Finally, the most heat-sensitive developmental stage was identified by observing the timing of the lowest pollen viability.

## Materials and methods

### Plant materials

A total of 16 *Chenopodium* accessions were used for the field experiment in this study, categorized into five groups: *C. berlandieri* (south-west USA; four accessions); *C. hircinum* Argentina (three accessions); *C. hircinum* Chile (one accession); *C. quinoa* lowland ecotype (Chile, Argentina; four accessions); and *C. quinoa* highland ecotype (Bolivia, Peru; four accessions) (Supplementary Table S1). One representative accession of each group was used for further controlled growth-chamber experiments to compare pollen viability and seed set, to determine the most heat-sensitive stage of pollen development, and to test *in vitro* pollen germinability.

### Crop management in the field experiment

The experiment was conducted at the KAUST field site in Saudi Arabia (22°18′12.8ʺN, 39°06′40.9ʺE) during the 2022–2023 growing season. Seeds of the wild accessions were soaked for 48 h at 4 °C in 500 µM gibberellic acid (GA_3_; G7645, Sigma), followed by scarification under a dissection microscope. The scarified seeds were then spread on 90 mm filter paper (1004-090 Whatman) wetted with the GA_3_ solution in a Petri dish. Seeds of cultivated quinoa were not scarified and were directly spread on filter paper wetted with GA_3_. The Petri dishes were kept at room temperature for germination. Once seeds had produced a radicle of length 0.5 cm, they were shallowly covered in potting soil (Basissubstrat2, Stender) and placed in a greenhouse where the temperature was maintained at 25/20 °C day/night. The seedlings were exposed natural sunlight with supplementary lighting from LED lamps (200–1000 µmol s^−1^ m^−2^).

After 2 weeks, the seedlings were moved outside to natural conditions and transplanted to the field after 7 d acclimation. Each plot (1×1 m) contained 40 seedlings arranged in four rows with 10 plants per row (10 cm between plants, 30 cm between rows). A thin layer of compost and 33.3 g of 15-15-15 (N-P-K) complete fertilizer was spread evenly over the sandy soil along the row, and the field was irrigated daily. During flowering time, plants were fertilized with urea (46%) at a rate of 15.2 g per plot. The final rates for N, P, and K were equivalent to 120, 50, and 50 kg ha^–1^, respectively.

The experiment was split into three planting dates, with P1 aimed at the period of optimum temperature at flowering time, P2 aimed at moderate heat, and P3 aimed at extreme heat, and was designed as a split random complete block design with four replicate blocks. Within each block, the genotypes were randomly assigned to plots and planting dates were randomly assigned as sub-plots. In an attempt to synchronize flowering time among the different accessions, the sowing and transplanting dates of the accessions were staggered. The transplanting for P1 was done between 2nd November 2022 and 3rd January 2023, for P2 it was between 2nd February and 13th March 2023, and for P3 it was between 18th June and 5th July 2023.

### Phenotypic characterization of plants in the field experiment

For each plot, days to first flowering and days to harvest were recorded. When plants were at the stage of 50% flowering, three fully expanded leaves at the top of canopy directly facing sunlight were selected in each plot for stomatal conductance measurements with a LI-600 porometer (LI-COR Environmental). The temperatures of three fully expanded leaves at the top of canopy and two panicles per plot were also measured, using an infrared thermometer (TFI550, Ebro, Xylem Analytics Germany GmbH). The porometer and infrared thermometer measurements were taken between 09.00 h and 11.00 h on full-sunlight days. A SPAD-502Plus chlorophyll meter (Konica Minolta) was used to assess the chlorophyll content every week on the same three leaves per plot from 50% flowering through to senescence. To address the issue of seed shedding of wild relatives during ripening, four plants per plot were bagged after flowering and harvested at maturity. Plant height at maturity was recorded. After harvesting, the material from each of the four bagged plant was dried at 40 °C for 1 week, followed by manual threshing. The seeds were cleaned with a blower and weighed to determine the yield per plant. Using an automated counter, samples of ∼1000 grains were weighed to determine the thousand-grain weight.

The accumulated thermal times from sowing to flowering (GDDfl) and from flowering to maturation (GDDrgf) were determined as growing degree-days with base temperature of 3 °C.

### Chamber experiment for pollen viability and seed setting

The pollen viability of one representative accession from each of the five groups (Supplementary Table S1) was examined in a temperature-controlled chamber. All seeds were soaked in 500 µM GA_3_ solution for 48 h at 4 °C and then those of the wild relatives were scarified with a sharp scalpel to remove the seed coat around the embryo. The scarified seeds of the wild relatives and the non-scarified seeds of the cultivated quinoa were then incubated on wet filter paper placed in Petri dishes for 3–4 d. The seedlings were then transplanted into plastic pots filled with a mixture of sand, gravel, and potting soil (Basissubstrat2) at a ratio of 2:1:1, and transferred to a greenhouse under the conditions described above.

When the first inflorescence was visible (growth stage BBCH 50-51; Sosa-Zuniga *et al.*, 2017), the plants were moved to a growth chamber (PGC-20L1, Percival Scientific, Inc.). The temperature in this control chamber was 25/20 °C day/night, with a 12/12 h photoperiod and light intensity at the canopy level of 400 μmol m^−2^ s^−1^, and relative humidity maintained at ∼70%. Rather than abrupt transitions, the temperature and light intensity were increased over a 3 h period (6-9 a.m.)at the night/day transition and decreased over a 3 h period (3-6 p.m.) at the day/night transition. From the second day after observing the first open flower, the freshly opened flowers on each day were labelled using permanent markers with different colors (Supplementary Fig. S1). On the third labelling day, the plants were moved into another chamber for heat stress, with the temperature set at 38/33 °C day/night and all other settings kept the same. The heat stress was maintained for 5 d, after which the plants were moved back to the control chamber. Labelling of the opened flowers was continued during and after the heat-stress treatment for a total duration of 19 d. This method enabled us to track the anthesis dates of the flowers, and therefore to examine the heat effects on seed set at different developmental stages. At 2 weeks after the last labelling, all the flowers were harvested and separated according to the different colors with which they were marked. Seed set was calculated as the number of seeded flowers divided by the total number of flowers. For pollen viability, pollen was collected daily from freshly opened flowers from the start of the heat-stress treatment and continued for 16 d. Pollen was stained for the presence of starch (Castillo *et al*., 2022) using Lugol’s solution: (1% w/v potassium iodide and 1% w/v iodine; both Sigma). Round, dark-stained pollen was considered as viable, while irregular-shaped and unstained pollen were deemed non-viable. Viability was calculated as the number of viable pollen divided by the total number of pollen, and expressed as a percentage. The pollen was counted using ImageJ (Schneider *et al.*, 2012) according to the method described by Tello *et al*. (2018). For both seed set and pollen viability, the developmental stages at the start of heat stress were expressed as days before flowering (Supplementary Fig. S1).

### Determination of pollen developmental stages in relation to days before flowering

To determine the developmental stages of the pollen in the growth chamber experiment described above so that they could be matched with days before flowering, buds at various pre-anthesis stages were labeled with permanent markers, and a single anther from each was sampled to determine its pollen developmental stage, leaving the other anthers untouched so that they could progress to anthesis (Supplementary Fig. S2). In total, seven pollen developmental stages were identified (Supplementary Fig. S3). The anthesis dates of the sampled flower buds were recorded so that the developmental stages of the pollen could be related to the stage expressed as days before flowering

### *In vitro* pollen germination and growth assays

To determine the effects of heat stress on pollen germination and growth, plants of the same five accessions used for the pollen viability analysis (Supplementary Table S1) were cultivated in the growth chamber under the control conditions described above. Mature pollen was collected from freshly opened flowers and suspended in germination medium, which was optimized for each accession (Supplementary Table S2). For each accession, three hanging drops of medium each containing pollen collected from at least three plants were placed inside a moisturized Petri dish, and the dishes were incubated in the dark for 24 h in an Isotemp I601D Incubator (Fisher Scientific) at temperatures varying from 22 °C to 40 °C at 2 °C intervals, for *in vitro* assessment of germination (Castillo *et al.*, 2022; Morris, 2022). The experiment was repeated three times on consecutive days. Pollen grains were considered germinated when the tube length was at least equal to or greater than the grain diameter. The number of germinated grains per field of view was divided by the total number of grains and expressed as a percentage. Depending on the amount of pollen available, 150–1700 grains were analysed for each replicate. In addition, pollen-tube elongation was measured at 34 °C and 38 °C using ImageJ (Schneider *et al.*, 2012). Mean pollen-tube length (PTL) was determined from measurements of at least 15 tubes in each of the three repeated experiments.

### Statistical analyses

For the field experiment, yield per plant, thousand-grain weight, grain number per plant and other physiological traits were analysed using linear mixed models (equation 1), where group (*Grp*) and planting date (*Pdate*) were treated as fixed factors, and accession (*g_i_*) and block (*b_j_*) as random effects:


(1) $$\begin{array}{rl} Y_{ijk}= & \;\beta_{0}+\beta_{1}\times Grp_{i}+\beta_{2}\times Pdate_{j}\\ & +\;\beta_{3}\times (Grp_{i}\times Pdate_{j})+g_{i}+b_{j}+\epsilon_{ijk} \end{array}$$

The models were implemented with *lmer* from the *lmerTest* package (Kuznetsova *et al.*, 2017). For pollen viability and seed set, the data of each representative accession was fitted against the splines of developmental stages (expressed as days before flowering, DBF) for the control and heat treatments, and the treatment was added as a linear term in the general additive models (equation 2), where *β_j_* represents the main effect of treatment *j*, and *f_j_*(*DBF*) is the smooth function of DBF for treatment *j*:


(2) $$Y=\beta_{0}+\beta_{\;j}+f_{\;j}(DBF)$$

The models were implemented through the *gam* function from the *mgcv* package (Wood, 2011). To find the most heat-sensitive flower bud developmental stage at which anthers had their lowest pollen viability, the derivatives of the fitted models were calculated with *derivatives* from the *gratia* package (Simpson, 2024). The lowest pollen viability is the *x*-axis coordinate (DBF) when the derivative equals zero.

*In vitro* pollen germination data for each representative accession were fitted to temperature-response curves using generalized additive models (equation 3) to estimate the maximum germination rate and the optimal temperature for germination:


(3) $$Y=\beta_{0}+f(Temperature)$$

This was calculated through the *gam* function from the *mgcv* package. Pollen-tube length data at 34 °C and 38 °C were analysed using two-way ANOVA, with accession and time as fixed factors, followed by Tukey’s HSD to determine significant differences. The analysis was implemented using the R software (v4.4.1; www.r-project.org).

## Results

### Reduction in grain number underlies yield loss under extreme reproductive heat stress

**Fig. 1.**
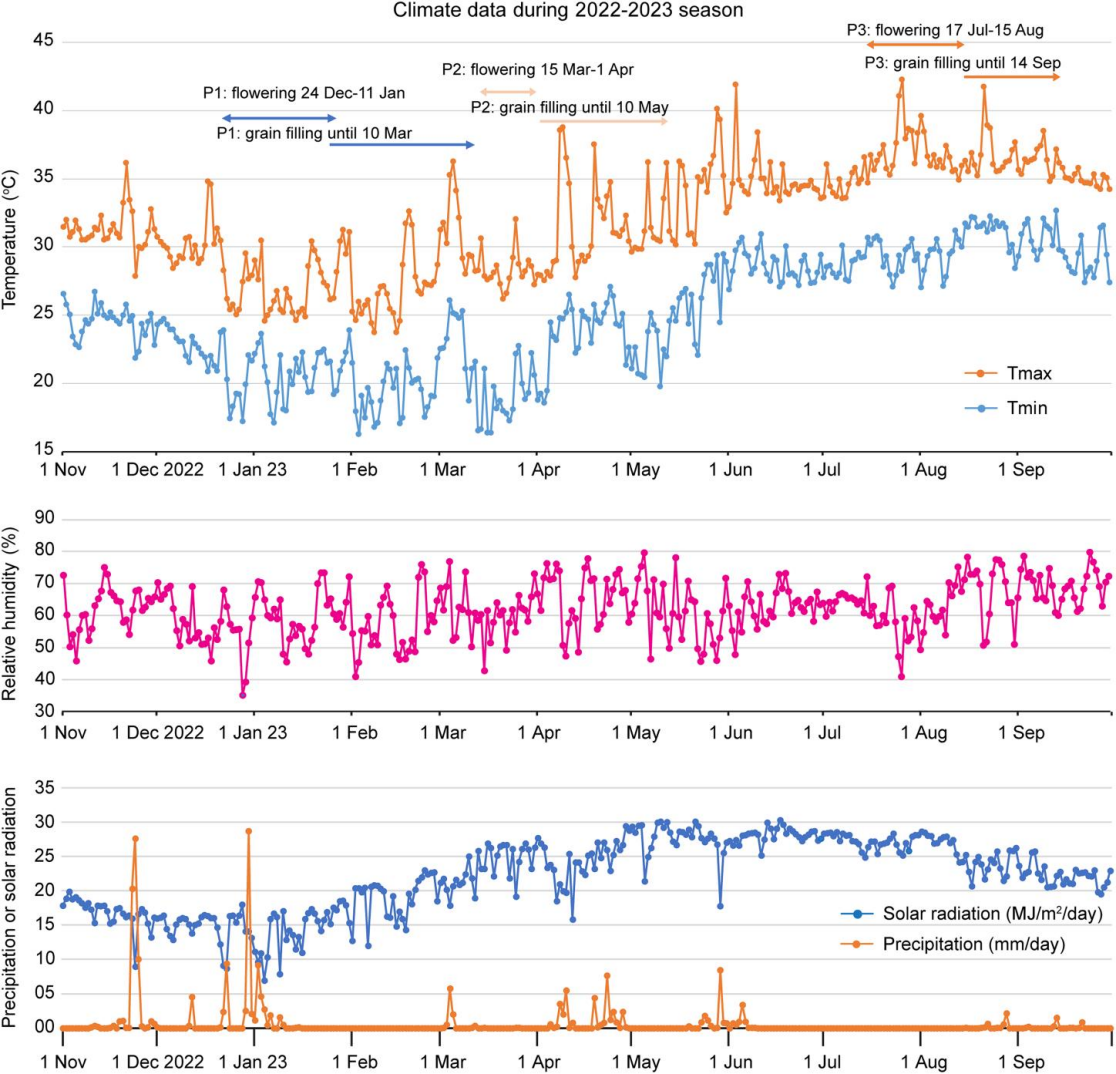
Climate data for the field experiment in the 2022–2023 season. Daily data are shown for maximum and minimum temperature (Tmax and Tmin), relative humidity, solar radiation, and precipitation. The flowering and grain-filling periods for the three planting dates (P1–P3) are indicated. For P1, the temperatures can be considered as optimal; for P2, they represent moderate heat stress; and for P3, they represent extreme heat stress.

The diversity panel of quinoa and its wild relatives were subjected to different levels of heat stress in the field as a consequence of different planting dates (P1–P3; Fig. 1). For P1, the flowering period was between 24^th^ December 2022 and 22^nd^ January 2023 and the temperature was around the optimal range for plant growth, with only rare occurrences over 30 °C. For P2, there was a moderate increase in temperature, especially during the grain-filling stage when temperatures were consistently above 30 °C and occasionally >35 °C. For P3, plants were exposed to extreme heat during their entire life cycle and the temperature was consistently above 35 °C. Solar radiation during the experiment was in the range 10–30 MJ m^–2^ d^–1^ and was highest between May and July, which was earlier than the highest values of temperature. Relative humidity fluctuated between 40–80% across the experiment with no evident trends. Rainfall was generally low except for two days between November and January.

**Fig. 2.**
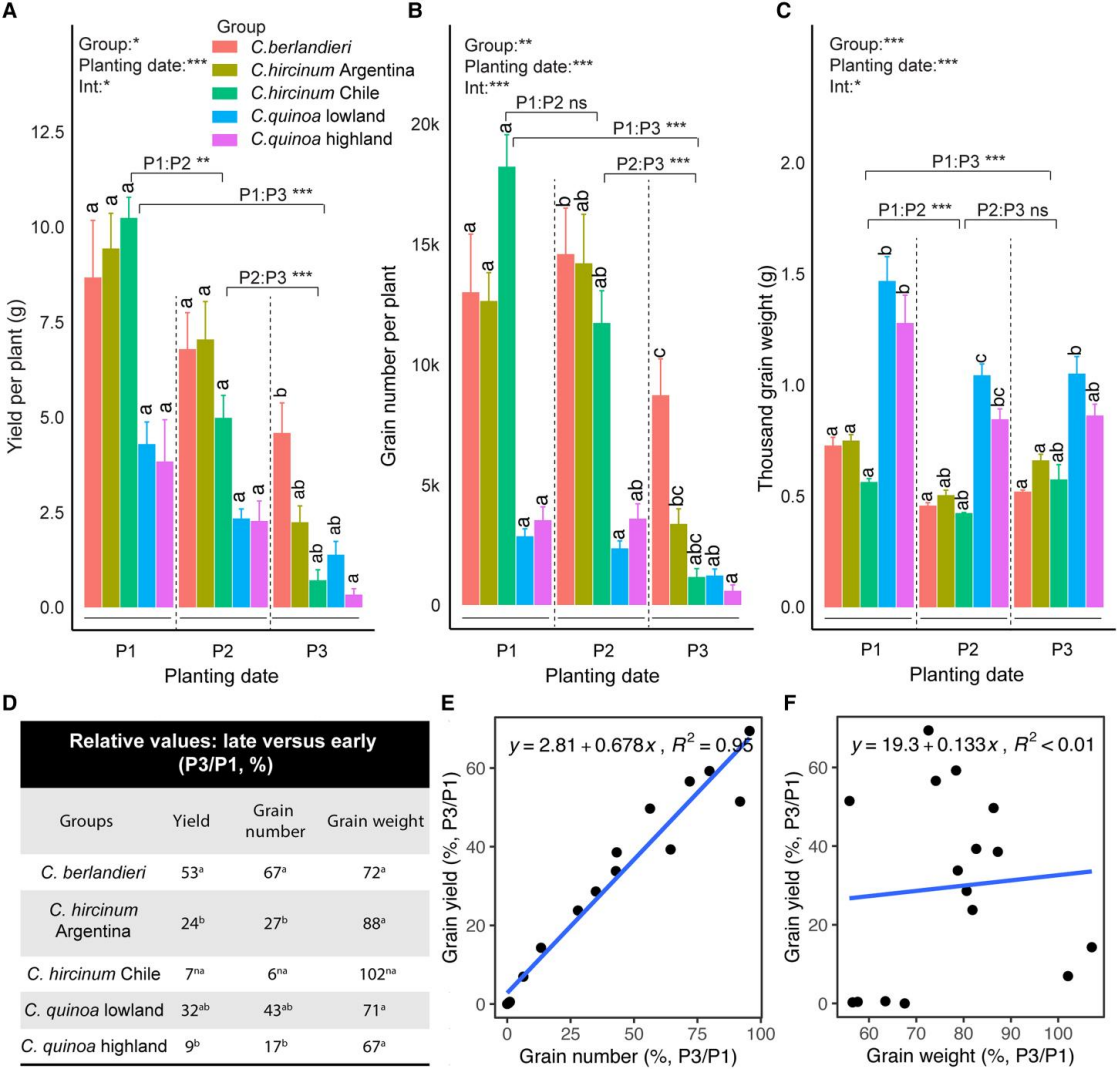
Effects of heat stress on yield, grain number, and grain weight of quinoa accessions in the field experiment. The planting dates P1, P2, and P3 represent optimal conditions, moderate heat stress, and extreme heat stress, respectively (Fig. 1). *Chenopodium quinoa* lowland and highland represent cultivated accessions whilst the other groups are wild relatives. (A) Grain yield per plant, (B) grain number per plant, and (C) thousand grain weight. Data are means (±SE) for 1–4 accessions in each group planted in four replicate blocks (see Supplementary Table S1). The results of two-way ANOVA to determine the effects of group, planting date, and their interactions (Int.) are shown, together with pairwise comparisons for the planting dates: ***P<0.001; **P<0.01; *P<0.05; ns, not significant. In addition, different letters indicate significant differences among means within individual planting dates, as determined using Tukey’s HSD test (*P*<0.05). (D) Yield, grain number, and grain weight in P3 relative to P1 (P3/P1) expressed as a percentage for each of the five groups of accessions. Different letters indicate significant differences among means as determined using Tukey’s HSD test (*P*<0.05). *Chenopodium hircinum* Chile contained only one accession and hence no statistical comparisons could be made (na, not applicable). (E) The relationship between the relative P3/P1 yield and grain number and (F) the relative P3/P1 yield and grain weight.

The yields of all five groups of accessions in P2 were significantly reduced compared with P1, and even more severe reductions were for P3 (Fig. 2A). In comparison with the other accessions at the hottest temperatures (P3), *C. berlandieri* was the highest yielding and had the highest grain number (Fig. 2B), whilst the *C. quinoa* highland accessions were the lowest yielding and had the lowest grain number. Grain size measured as thousand-grain weight (TGW) was reduced under the hotter conditions of P2 and P3 in comparison with P1, but the extreme heat conditions of P3 did not cause any further reductions relative to P2 (Fig. 2C). In comparison with P1, the grain number in P2 was only slightly reduced, probably due to the relatively small difference in temperatures during their flowering periods. In contrast, grain number was substantially decreased in P3, where the temperature was consistently over 35 °C during flowering (Fig. 1). Examination of the relative performance of P3 and P1 (P3/P1) showed that the values of yield and grain number well associated with each other (Fig. 2D), indicating that maintaining grain number was a key factor in sustaining the yield of the wild relative *C. berlandieri* under reproductive heat stress (Fig. 2E, F).

In P1 and P2 conditions, both yield and grain number were positively correlated with days to flowering, but this correlation was lost in plants grown in P3 conditions (Supplementary Tables S3–S5). The impact of the heat stress was clear in P3, as indicated by negative correlations between both yield and grain number and the thermal sum from sowing to flowering (GDDfl). In addition, stomatal conductance was positively associated with yield and grain number only under P3 conditions.

### Pollen viability limits seed set and hence grain number in cultivated quinoa

**Fig. 3.**
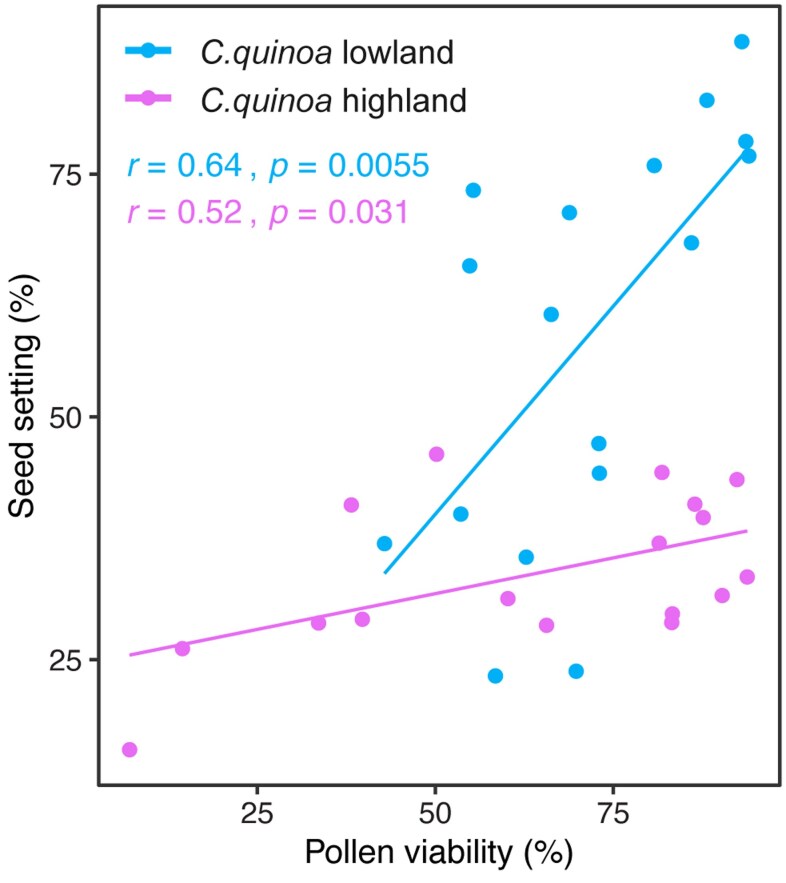
The relationship between seed setting and pollen viability in lowland (CQ1) and highland (CQ8) quinoa (see Supplementary Table S1). Plants were subjected to day/night heat stress in a growth chamber at 38/33 °C for 5 d and then returned to control conditions, and pollen viability was determined daily from the first day of treatment for a total of 17 d. Seed set was determined ∼4 weeks later. Each dot represents the daily mean values of at least three independent plants. Corresponding results for wild relatives are given in Supplementary Fig. S4.

Given its importance to yield, we further examined the physiological traits contributing to grain number in a growth chamber experiment. Pollen quality has been widely considered as the major limiting factor for successful fertilization (Zinn *et al.*, 2010; Xu *et al.*, 2017), and therefore we analysed the effects of heat (5 d at 38/33 °C, day/night) applied at different developmental stages on pollen viability and seed setting. A significant positive correlation was found between these two traits in both lowland and highland *C. quinoa* (Fig. 3), but not in the wild relatives (Supplementary Fig. S4). This suggested that pollen viability limited seed set in the cultivated accessions, and this might consequently have affected the number of grains under heat treatment.

### Wild *C. berlandieri* produces more viable pollen under heat stress

**Fig. 4.**
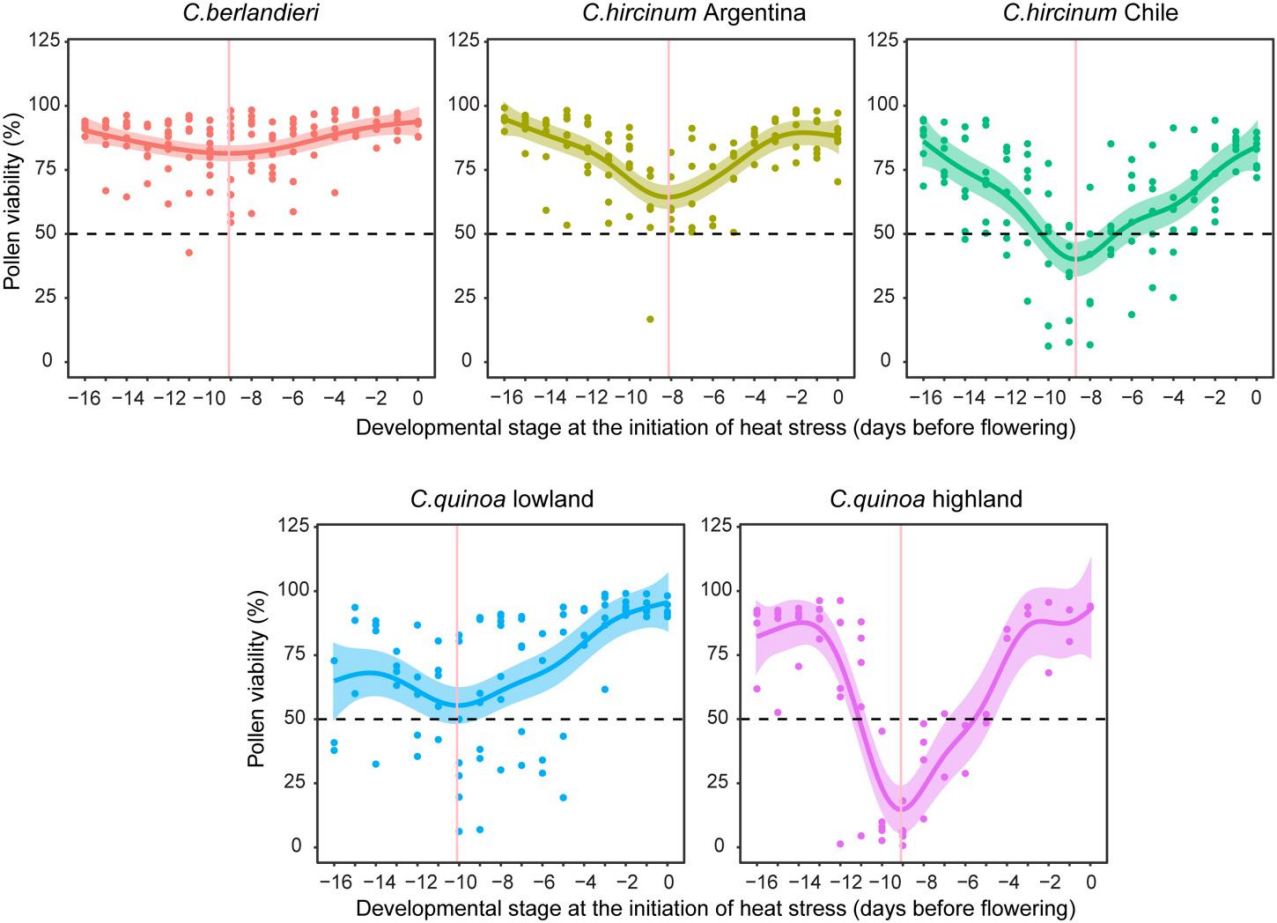
Variation in pollen viability among cultivated quinoa and wild relatives under heat stress. Plants of representative accessions (see Supplementary Table S1) were subjected to day/night heat stress in a growth chamber at 38/33 °C for 5 d and then returned to control conditions, and pollen viability was determined daily from the first day of treatment for a total of 17 d. The pollen viability data are fitted against days before flowering using general additive models (see Methods). For each accession, the dots represent the raw data, the dark line indicates the best-fitting curve, and the shading indicates the 95% confidence interval. The vertical lines indicate the point with the lowest modelled pollen viability. Corresponding results for control temperature conditions are given in Supplementary Fig. S5.

Under the control temperature (25/20 °C, day/night), pollen viability was maintained at a high level across consecutive developmental stages before flowering and it was similar among the representative accessions from each of the five groups (Supplementary Fig. S5). However, upon heat treatment (5 d at 38/33 °C, day/night), pollen viability decreased substantially, with considerable variation among the accessions. At its most heat-sensitive stage, *C. quinoa* highland showed only ∼10% pollen viability, while *C. berlandieri* maintained over 75% viability (Fig. 4). The overall heat tolerance of the five accessions could be ranked as *C. berlandieri* > *C. hircinum* Argentina > *C. quinoa* lowland > *C. hircinum* Chile > *C. quinoa* highland. Thus, wild *C. berlandieri* is heat tolerant during pollen development and could potentially be used as valuable resource for introgression of heat tolerance into cultivated quinoa, especially highland ecotypes.

### Early pollen mother cell is the most sensitive stage for imposition of heat

**Fig. 5.**
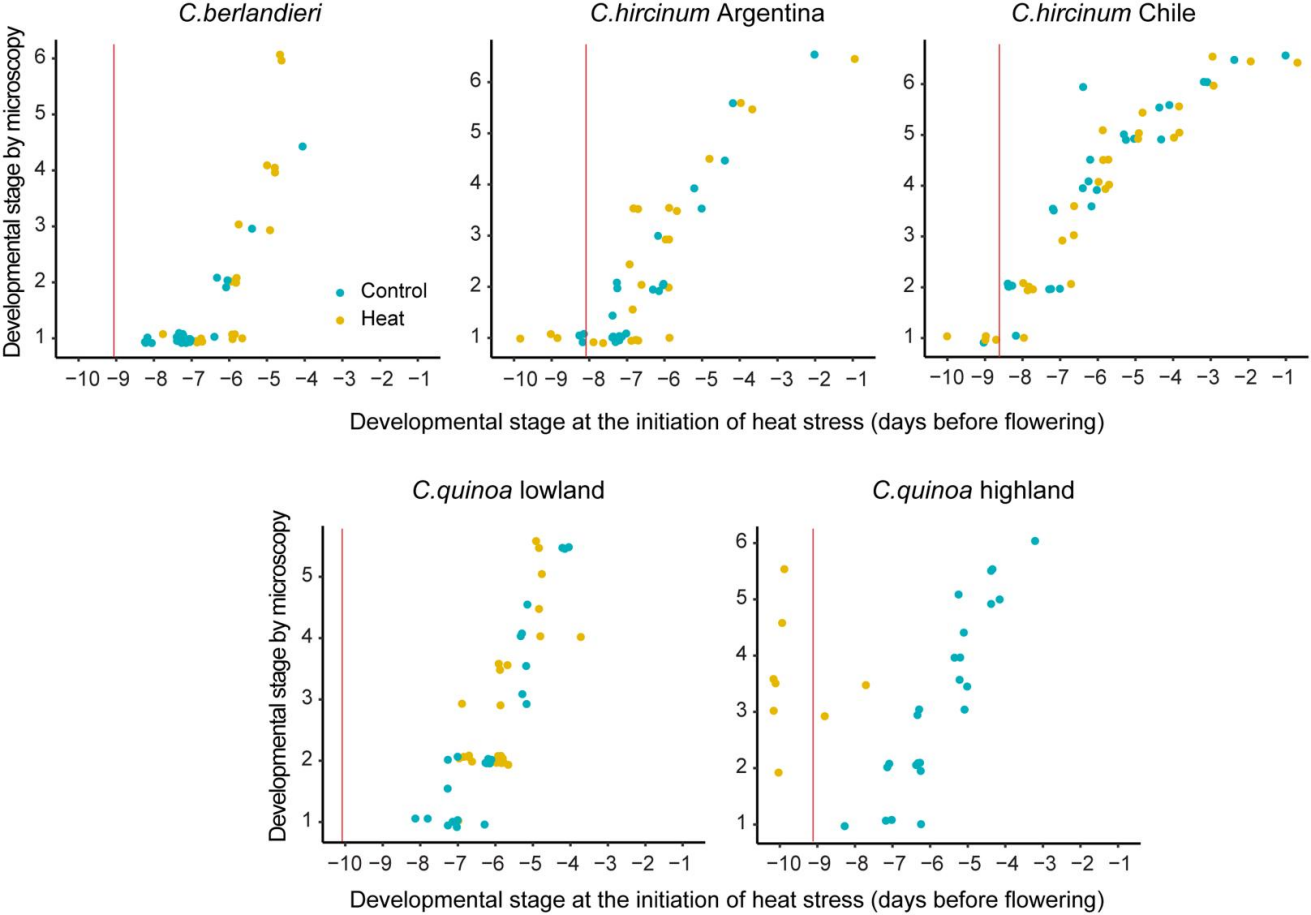
The mother cell stage of pollen development is the most sensitive to the imposition of heat stress in quinoa. Plants of representative accessions of cultivated quinoa and its wild relatives (see Supplementary Table S1) were subjected to day/night heat stress in a growth chamber at 38/33 °C for 5 d and then returned to control conditions (25/20 °C). DAPI staining and detailed microscopic examination were carried out in order to relate pollen development to the number of days before flowering at which the heat stress was imposed. The developmental stages by microscopy are as follows: 1, pollen mother cell; 2, tetrads; 3, newly released microspore; 4, early microspore; 5, polarized microspore; 6, bicellular microspore (see Supplementary Fig. S3). Each dot represents a pollen sample taken from an individual flower in the experiment. The vertical lines indicate the most heat-sensitive point for pollen viability as determined by modelling (Fig. 4), and all the accessions were at stage 1, pollen mother cell development. The relationships between the development stage and days before flowering were not affected by heat stress except for cultivated highland quinoa.

To identify the most heat-sensitive developmental stage (i.e. at the time when the lowest pollen viability was observed), we used DAPI staining and detailed microscopic examination in order to relate pollen development to the days before flowering at which the heat stress was imposed (Supplementary Fig. S2). Across all five accessions, seven developmental stages were identified: pollen mother cell, tetrads, newly released microspores, early microspores, polarized microspores, bi-cellular microspores, and mature tri-cellular pollen (Supplementary Fig. S3). For all the accessions, the most heat-sensitive stage was ∼8–10 d before flowering (Fig. 4), and in all cases the microscopic examination showed that this corresponded to the stage of early pollen mother cell development. Imposition of the heat treatment did not cause a significant deviation of the relationship between days before flowering and the pollen developmental stages, except in *C. quinoa* highland (Fig. 5). This was probably due to the formation of a lot of cleistogamous flowers, which interfered with the determination of exact flowering dates.

### Wild *C. berlandieri* is heat-tolerant during pollen germination and elongation

**Fig. 6.**
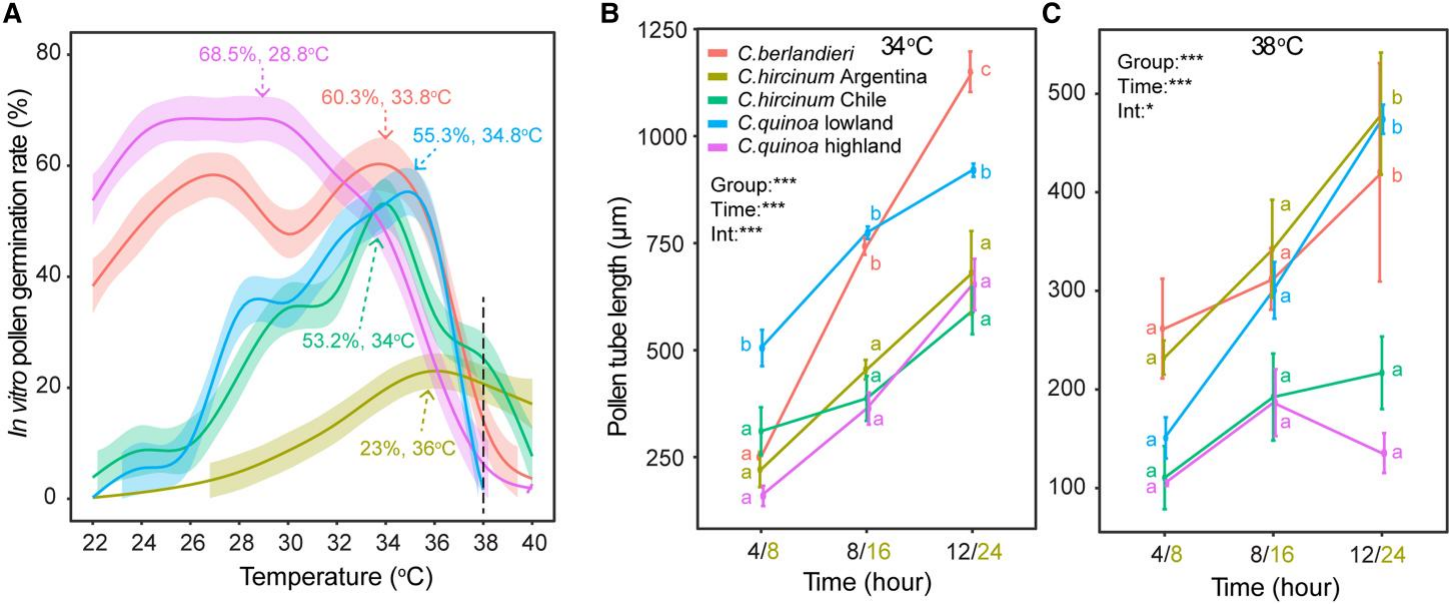
Variability of *in vitro* pollen germination at different temperatures in cultivated quinoa and its wild relatives. Pollen grains from plants of representative accessions (see Supplementary Table S1) were incubated in germination media at temperatures varying from 22 °C to 40 °C at 2 °C intervals. (A) The relationship between *in vitro* pollen germination rate and temperature, fitted using general additive models (see Methods). The maximum germination rate and corresponding optimum temperature as determined by the models are shown. (B, C) Pollen tube growth during incubation at (B) 34 °C and (C) 38 °C. For *C. hircinum* Argentina, measurements were taken at 8, 16, and 24 h; for the other accessions, measurements were taken at 4, 8, and 12 h. Data are means (±SE), *n*=3. The results of two-way ANOVA to determine the effects of group (accession), incubation time, and their interactions (Int.) are shown: ***P<0.001; *P<0.05. In addition, different letters indicate significant differences among means at the three time-points, as determined using Tukey’s HSD test (*P*<0.05).

We next examined the effects of a range of temperatures on *in vitro* pollen germination and pollen-tube growth. Both *C. berlandieri* and *C. quinoa* highland showed high germination rates (>60%) under a wide range of temperatures, but model-fitting indicated that germination of the latter began to decline at 28.8 °C, whereas germination of *C. berlandieri* was not affected until 33.8 °C (Fig. 6A). The other three accessions had relatively narrow ranges of temperature for high germination, and they were at higher temperatures (36, 34.8, and 34 °C for *C. hircinum* Argentina, *C. quinoa* lowland, and *C. hircinum* Chile, respectively). At 38 °C, the wild relatives all showed better germination than lowland and highland quinoa. For pollen-tube elongation at 34 °C, there were highly significant effects of both group (accession) and incubation time, as well as their interaction (Fig. 6B). The pollen tubes of *C. berlandieri* were the longest after 12 h of incubation, followed by *C. quinoa* lowland. For pollen-tube elongation at 38 °C, there were also significant effects for group, incubation time, and their interaction (Fig. 6C). After 12 h of incubation, the longest pollen tubes were observed in *C. berlandieri*, *C. hircinum* Argentina, and *C. quinoa* lowland. Overall, *C. berlandieri* performed comparatively well in terms of its absolute maximum germination rate, optimum temperature for maximum germination, and germination rate and pollen tube elongation at 38 °C.

## Discussion

Heat stress is considered as one of the main factors limiting the expansion of quinoa cultivation and threatening its production under forecasted climate change scenarios (Jellen *et al.*, 2013). In the present study, different degrees of heat stress were applied in the field via the use of different planting dates (Fig. 1). Under moderate heat during the grain-filling stage, grain size was the major factor underlying yield reductions (Fig. 2), whereas under extreme heat during flowering a large reduction was observed in grain number. Notably, wild *C. berlandieri* maintained higher yields than cultivated quinoa due to better maintenance of grain number. This observation confirms the contribution of grain number to yield under heat stress, consistent with previous studies (Lesjak and Calderini, 2017; Tovar *et al.*, 2020), and led us to explore the role of pollen viability, which was positively correlated with seed set in both highland and lowland quinoa under heat treatment (Fig. 3). This association has been previously reported in other crops under heat stress (Jagadish *et al.*, 2007; Xu *et al.*, 2022), suggesting a common and important role of pollen viability in seed set. Using developmental stage-specific and temperature-controlled experiments, we observed a clear difference in pollen viability between cultivated quinoa and wild *C. berlandieri* (Fig. 4). Therefore, it seems likely that the yield advantage of wild *C. berlandieri* over cultivated quinoa under high temperature conditions was achieved through production of more viable pollen, making it a key trait to improve the heat tolerance of cultivated quinoa (Allaoui *et al.*, 2023; Maughan *et al.*, 2024). The fact that *C. berlandieri* is distributed throughout hot desert environments in southwestern USA, where it flowers and sets seed in the summer season (June–September), suggests that there should be an abundance source of heat-tolerant genes for improving quinoa through wide interspecies crossing (Jellen *et al.*, 2019). Introduction of heat tolerance from *C. berlandieri* to cultivated quinoa should be feasible as fertile hybrids, mapping populations, and faithful recombinations have been generated between them (Allaoui *et al.*, 2023; Maughan *et al.*, 2024), and the availability of genetic information for *C. berlandieri* should facilitate marker- or genomic-assisted selection (Maughan *et al.*, 2024), enabling fast recovery of desirable agronomic traits after crossing with cultivated quinoa.

Heat tolerance is a rather complex trait to try to understand by looking at yield alone under high temperatures, and focusing instead on sub-traits that contribute to yield with a top-down approach enabled us to identify key traits that can be more easily selected and are genetically tractable (Morton *et al.*, 2019; Reynolds *et al.*, 2020), and which could be combined to improve overall heat tolerance. In addition to pollen viability, other factors such as female fertility and early seed development also limit seed set under heat stress. Female fertility is related to ovule viability and the presence of pollen-receptive stigmas under heat stress (Hedhly *et al.*, 2003; Wang *et al.*, 2021; Shi *et al.*, 2022), and further investigations are needed to examine its effects in quinoa using male-sterile lines. Phenological traits are very adaptive to heat and large variations has been observed in both cultivated quinoa (Rahman *et al.*, 2024) and its wild relatives (Curti *et al.*, 2022), indicating the possibility of selecting early-maturing genotypes to avoid the effects of heat on reproductive development and grain filling (Matías *et al.*, 2021). Wild relatives also tend to be more developmentally indeterminate, with side-branches continuously generated from the lower part of the stem. This developmental asynchrony could be a strategy evolved by plants to escape short periods of heat (Curti *et al.*, 2022). Taken together, these traits and their underlying mechanisms need to be precisely phenotyped (Stanschewski *et al.*, 2021), genetically dissected, and then integrated with agronomic practices in quinoa cultivation to improve crop performance.

Given the importance of pollen viability in seed setting under heat stress, reliable high-throughput methods are needed for its quantification. Specifically for quinoa, pollen viability has mainly been analysed with staining methods that capture different properties of viable pollen, such as Alexander staining (Tovar *et al.*, 2020), triphenyl tetrazolium chloride (TTC) staining (Hinojosa *et al.*, 2019), and Lugol’s staining for the presence of starch, as well as *in vitro* germination assays (Castillo *et al.*, 2022; Morris, 2022). These methods generally deal with a few hundred pollen grains and largely rely on image processing and analysis. Staining methods often give false-positive results, and the germination medium has to be optimized for different pollen, even at the level of different quinoa varieties (e.g. Supplementary Table S2). As an alternative, impedance flow cytometry, which relies on electro-magnetic properties to determine viability, has emerged as a promising assessment platform (Heidmann *et al.*, 2016), and has been successfully with a wide range of crops. Its advantages over staining and *in vitro* germination assays are greater reliability, as it analyses at least 10 000 grains, and speed, thereby significantly reducing the analysis time and resulting in higher throughput. Establishing this platform to examine pollen viability of quinoa and its wild relatives would be worthwhile.

In conclusion, our study demonstrated that wild relatives of quinoa, and particularly *C. berlandieri*, exhibited higher yield stability than cultivated accessions under high temperatures in field conditions, and this was primarily due to their ability to maintain higher grain numbers. We also found that pollen viability limited seed setting in cultivated quinoa, as indicated by positive correlations observed between these traits. Detailed examination of cultivated quinoa and its wild relatives showed that *C. berlandieri* clearly had the best ability to maintain high pollen viability under heat stress, which most likely explained its subsequent higher grain numbers and yield. Hence, *C. berlandieri* could be a very valuable genetic resource for introgressing heat tolerance into cultivated quinoa.

## Supplementary Material

eraf235_Supplementary_Data

## Data Availability

All primary data that support the findings of this study are openly available at Dryad Digital Repository https://doi.org/10.5061/dryad.d7wm37qbx; Xu *et al.*, (2025).

## Supplementary data

The following supplementary data are available at *JXB*  online.

Fig. S1. Illustration of the scheme used for analysis of seed setting and pollen viability.

Fig. S2. Illustration of the scheme used to relate days before flowering with the pollen developmental stage as determined by microscopy.

Fig. S3. The seven developmental stages identified during pollen development.

Fig. S4. Relationships between seed setting and pollen viability for the three wild relatives.

Fig. S5. Pollen viability of cultivated quinoa and its wild relatives under the control temperature treatment.

Table S1. Details of all the accessions used in this study.

Table S2. Details of the different optimized media used for *in vitro* pollen germination assays for each accession.

Table S3. Correlation matrix for plant and environmental parameters for planting date 1.

Table S4. Correlation matrix for plant and environmental parameters for planting date 2.

Table S5. Correlation matrix for plant and environmental parameters for planting date 3.

## References

[eraf235-B1] Abbas G, Murtaza B, Amjad M, Saqib M, Akram M, Naeem MA, Shah GM, Raza M, Ali Q, Ahmed K. 2024. Heat stress resulting from late sowing impairs grain yield and quality of quinoa genotypes facing drought and salt stress under field conditions. Journal of Agronomy and Crop Science 210, e12717.

[eraf235-B2] Alandia G, Rodriguez JP, Jacobsen SE, Bazile D, Condori B. 2020. Global expansion of quinoa and challenges for the Andean region. Global Food Security 26, 100429.

[eraf235-B3] Allaoui A, Jellen EN, Thiam EH, Benlhabib O. 2023. Evaluation of *Chenopodium quinoa* × *C. berlandieri* recombinant inbred lines (RILs) for heat tolerance. Chilean Journal of Agricultural Research 83, 260–271.

[eraf235-B4] Alvar-Beltrán J, Verdi L, Marta AD, Dao A, Vivoli R, Sanou J, Orlandini S. 2020. The effect of heat stress on quinoa (cv. Titicaca) under controlled climatic conditions. The Journal of Agricultural Science 158, 255–261.

[eraf235-B5] Bazile D, Jacobsen SE, Verniau A. 2016. The global expansion of quinoa: trends and limits. Frontiers in Plant Science 7, 622. 27242826 10.3389/fpls.2016.00622 PMC4860459

[eraf235-B6] Begcy K, Nosenko T, Zhou LZ, Fragner L, Weckwerth W, Dresselhaus T. 2019. Male sterility in maize after transient heat stress during the tetrad stage of pollen development. Plant Physiology 181, 683–700. 31378720 10.1104/pp.19.00707 PMC6776839

[eraf235-B7] Castillo SE, Tovar JC, Shamin A, Gutirerrez J, Pearson P, Gehan MA. 2022. A protocol for *Chenopodium quinoa* pollen germination. Plant Methods 18, 65. 35585546 10.1186/s13007-022-00900-3 PMC9118578

[eraf235-B8] Coughlan de Perez E, Ganapathi H, Masukwedza GIT, Griffin T, Kelder T. 2023. Potential for surprising heat and drought events in wheat-producing regions of USA and China. npj Climate and Atmospheric Science 6, 56. 38665270 10.1038/s41612-023-00361-y PMC11041665

[eraf235-B9] Curti RN, Ortega-Baes P, Ratto S, Bertero D. 2022. Harnessing phenological traits of wild ancestor *Chenopodium hircinum to* improve climate adaptation of quinoa. Crop & Pasture Science 74, 1058–1068.

[eraf235-B10] FAO & CIRAD . 2013. State of the art report of quinoa in the world in 2013. Bazile D, Bertero D, Nieto C, eds. Rome: FAO.

[eraf235-B11] Hedhly A, Hormaza JI, Herrero M. 2003. The effect of temperature on stigmatic receptivity in sweet cherry (*Prunus avium* L.). Plant, Cell & Environment 26, 1673–1680.

[eraf235-B12] Heidmann I, Schade-Kampmann G, Lambalk J, Ottiger M, Di Berardino M. 2016. Impedance flow cytometry: a novel technique in pollen analysis. PLoS ONE 11, e0165531. 27832091 10.1371/journal.pone.0165531 PMC5104384

[eraf235-B13] Hinojosa L, Matanguihan JB, Murphy KM. 2019. Effect of high temperature on pollen morphology, plant growth and seed yield in quinoa (*Chenopodium quinoa* Willd.). Journal of Agronomy and Crop Science 205, 33–45.

[eraf235-B14] IPCC . 2023. AR6 synthesis report. Climate change 2023. Geneva: IPCC

[eraf235-B15] Ishimaru T, Hirabayashi H, Ida M, Takai T, San-Oh YA, Yoshinaga S, Ando I, Ogawa T, Kondo M. 2010. A genetic resource for early-morning flowering trait of wild rice *Oryza officinalis* to mitigate high temperature-induced spikelet sterility at anthesis. Annals of Botany 106, 515–520. 20566680 10.1093/aob/mcq124 PMC2924824

[eraf235-B16] Jagadish S, Craufurd P, Wheeler T. 2007. High temperature stress and spikelet fertility in rice (*Oryza sativa* L.). Journal of Experimental Botany 58, 1627–1635. 17431025 10.1093/jxb/erm003

[eraf235-B17] Jarvis DE, Ho YS, Lightfoot DJ, et al 2017. The genome of *Chenopodium quinoa*. Nature 542, 307–312. 28178233 10.1038/nature21370

[eraf235-B18] Jellen EN, Jarvis DE, Hunt SP, Mangelsen HH, Maughan PJ. 2019. New seed collections of North American pitseed goosefoot (*Chenopodium berlandieri*) and efforts to identify its diploid ancestors through whole-genome sequencing. Ciencia e Investigación Agraria 46, 187–196.

[eraf235-B19] Jellen EN, Maughan PJ, Fuentes F, Kolano B. 2013. Botany, phylogeny and evolution. In: FAO & CIRAD. State of the art report of quinoa in the world in 2013. Rome: FAO, 12–23.

[eraf235-B20] Kuznetsova A, Brockhoff PB, Christensen RHB. 2017. lmerTest package: tests in linear mixed effects models. Journal of Statistical Software 82, 1–26.

[eraf235-B21] Lesjak J, Calderini DF. 2017. Increased night temperature negatively affects grain yield, biomass and grain number in Chilean quinoa. Frontiers in Plant Science 8, 240631. 10.3389/fpls.2017.00352 PMC5362734 28386266

[eraf235-B22] Li XM, Chao DY, Wu Y, et al 2015. Natural alleles of a proteasome α2 subunit gene contribute to thermotolerance and adaptation of African rice. Nature Genetics 47, 827–833. 25985140 10.1038/ng.3305

[eraf235-B23] Luo N, Mueller N, Zhang Y, Feng P, Huang S, Liu DL, Yu Y, Wang X, Wang P, Meng Q. 2023. Short-term extreme heat at flowering amplifies the impacts of climate change on maize production. Environmental Research Letters 18, 084021.

[eraf235-B24] Matías J, Rodríguez MJ, Cruz V, Calvo P, Reguera M. 2021. Heat stress lowers yields, alters nutrient uptake and changes seed quality in quinoa grown under Mediterranean field conditions. Journal of Agronomy and Crop Science 207, 481–491.

[eraf235-B25] Maughan PJ, Jarvis DE, de la Cruz-Torres E, et al 2024. North American pitseed goosefoot (*Chenopodium berlandieri*) is a genetic resource to improve Andean quinoa (*C. quinoa*). Scientific Reports 14, 12345. 38811833 10.1038/s41598-024-63106-8 PMC11137100

[eraf235-B26] Molero G, Coombes B, Joynson R, Pinto F, Piñera-Chávez FJ, Rivera-Amado C, Hall A, Reynolds MP. 2023. Exotic alleles contribute to heat tolerance in wheat under field conditions. Communications Biology 6, 21. 36624201 10.1038/s42003-022-04325-5 PMC9829678

[eraf235-B27] Morris A . 2022. Effect of heat stress on in vitro pollen germination and pollen tube elongation of Chenopodium quinoa and wild relatives. MS thesis, King Abdullah University of Science and Technology, Thuwal, Saudi Arabia. 10.25781/KAUST-1SJ0P.

[eraf235-B28] Morton MJL, Awlia M, Al-Tamimi N, Saade S, Pailles Y, Negrão S, Tester M. 2019. Salt stress under the scalpel – dissecting the genetics of salt tolerance. The Plant Journal 97, 148–163. 30548719 10.1111/tpj.14189 PMC6850516

[eraf235-B29] NOAA National Centers for Environmental Information . 2024. Annual 2024 global climate report. https://www.ncei.noaa.gov/access/monitoring/monthly-report/global/202413. Accessed 21 May 2025.

[eraf235-B30] Patiranage DSR, Rey E, Emrani N, Wellman G, Schmid K, Schmöckel SM, Tester M, Jung C. 2022. Genome-wide association study in quinoa reveals selection pattern typical for crops with a short breeding history. eLife 11, e66873. 35801689 10.7554/eLife.66873 PMC9388097

[eraf235-B31] Rahman H, Vikram P, Hu Y, et al 2024. Mining genomic regions associated with agronomic and biochemical traits in quinoa through GWAS. Scientific Reports 14, 9205. 38649738 10.1038/s41598-024-59565-8 PMC11035704

[eraf235-B32] Rey E, Maughan PJ, Maumus F, Lewis D, Wilson L, Fuller J, Schmöckel SM, Jellen EN, Tester M, Jarvis DE. 2023. A chromosome-scale assembly of the quinoa genome provides insights into the structure and dynamics of its subgenomes. Communications Biology 6, 1263. 38092895 10.1038/s42003-023-05613-4 PMC10719370

[eraf235-B33] Reynolds M, Chapman S, Crespo-Herrera L, et al 2020. Breeder friendly phenotyping. Plant Science 295, 110396. 32534615 10.1016/j.plantsci.2019.110396

[eraf235-B34] Satake T, Yoshida S. 1978. High temperature-induced sterility in indica rices at flowering. Japanese Journal of Crop Science 47, 6–17.

[eraf235-B35] Schneider CA, Rasband WS, Eliceiri KW. 2012. NIH image to ImageJ: 25 years of image analysis. Nature Methods 9, 671–675. 22930834 10.1038/nmeth.2089 PMC5554542

[eraf235-B36] Shi W, Yang J, Kumar R, Zhang X, Impa SM, Xiao G, Jagadish SVK. 2022. Heat stress during gametogenesis irreversibly damages female reproductive organ in rice. Rice 15, 32. 35763153 10.1186/s12284-022-00578-0 PMC9240181

[eraf235-B37] Simpson GL . 2024. gratia: An R package for exploring generalized additive models. The Journal of Open Source Software 9, 6962.

[eraf235-B38] Sosa-Zuniga V, Brito V, Fuentes F, Steinfort U. 2017. Phenological growth stages of quinoa (*Chenopodium quinoa*) based on the BBCH scale. The Annals of Applied Biology 171, 117–124.

[eraf235-B39] Stanschewski CS, Rey E, Fiene G, et al 2021. Quinoa phenotyping methodologies: an international consensus. Plants 10, 1759. 34579292 10.3390/plants10091759 PMC8472428

[eraf235-B40] Tello J, Montemayor MI, Forneck A, Ibáñez J. 2018. A new image-based tool for the high throughput phenotyping of pollen viability: evaluation of inter- and intra-cultivar diversity in grapevine. Plant Methods 14, 3. 29339970 10.1186/s13007-017-0267-2 PMC5759351

[eraf235-B41] Tovar JC, Quillatupa C, Callen ST, Castillo SE, Pearson P, Shamin A, Schuhl H, Fahlgren N, Gehan MA. 2020. Heating quinoa shoots results in yield loss by inhibiting fruit production and delaying maturity. The Plant Journal 102, 1058–1073. 31971639 10.1111/tpj.14699 PMC7318176

[eraf235-B42] Wahid A, Gelani S, Ashraf M, Foolad M. 2007. Heat tolerance in plants: an overview. Environmental and Experimental Botany 61, 199–223.

[eraf235-B43] Wang Y, Impa SM, Sunkar R, Jagadish SVK. 2021. The neglected other half - role of the pistil in plant heat stress responses. Plant, Cell & Environment 44, 2200–2210. 10.1111/pce.14067 33866576

[eraf235-B44] Wood SN . 2011. Fast stable restricted maximum likelihood and marginal likelihood estimation of semiparametric generalized linear models. Journal of the Royal Statistical Society: Series B, Statistical Methodology 73, 3–36.

[eraf235-B45] Xu J, Farooq H, Hashim M, et al 2025. Data from: Wild relatives to improve heat tolerance of cultivated quinoa (*Chenopodium quinoa* Willd.): pollen viability and grain number. Dryad Digital Repository. 10.5061/dryad.d7wm37qbx PMC12587419 40452430

[eraf235-B46] Xu J, Lowe C, Hernandez-Leon SG, Dreisigacker S, Reynolds MP, Valenzuela-Soto EM, Paul MJ, Heuer S. 2022. The effects of brief heat during early booting on reproductive, developmental, and chlorophyll physiological performance in common wheat (*Triticum aestivum* L.). Frontiers in Plant Science 13, 886541. 35651779 10.3389/fpls.2022.886541 PMC9149578

[eraf235-B47] Xu J, Wolters-Arts M, Mariani C, Huber H, Rieu I. 2017. Heat stress affects vegetative and reproductive performance and trait correlations in tomato (*Solanum lycopersicum*). Euphytica 213, 156.

[eraf235-B48] Young LA, Maughan PJ, Jarvis DE, et al 2023. A chromosome-scale reference of *Chenopodium watsonii* helps elucidate relationships within the North American A-genome *Chenopodium* species and with quinoa. The Plant Genome 16, e20349. 37195017 10.1002/tpg2.20349 PMC12807170

[eraf235-B49] Zinn KE, Tunc-Ozdemir M, Harper JF. 2010. Temperature stress and plant sexual reproduction: uncovering the weakest links. Journal of Experimental Botany 61, 1959–1968. 20351019 10.1093/jxb/erq053 PMC2917059

