# Supplementary material for: Wild relatives to improve heat tolerance of cultivated quinoa (Chenopodium quinoa): pollen viability and grain number
Source: J Exp Bot. 2025 Jun 2;76(17):5117–28. doi: 10.1093/jxb/eraf235 (PMC12587419; doi:10.1093/jxb/eraf235)
Supplement: eraf235_Supplementary_Data [file eraf235_supplementary_data.zip › jexbot314426-file001.pdf]

Supplementary Figures:

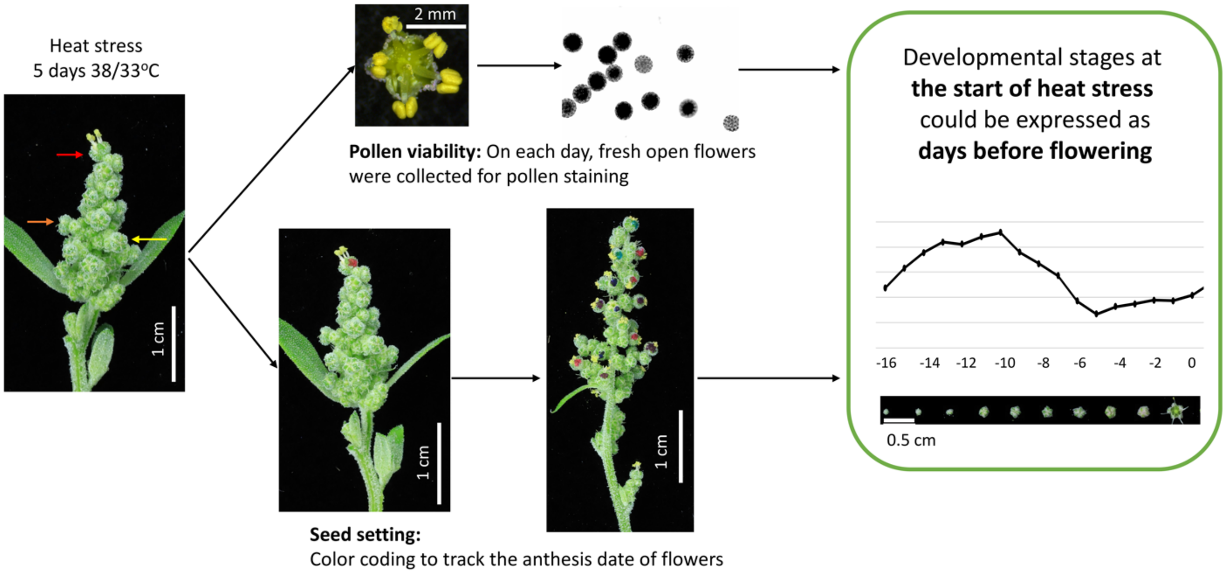

Fig S1. **Schematic illustration for seed setting and pollen viability analysis.** Around flowering, freshly opened flowers were harvested every day to collect pollen for viability analysis with staining for starch presence. As for seed setting analysis, freshly opened flowers were labelled with permanent markers with different colors (each color per day) to track the anthesis dates. Therefore, at the start of heat stress, developmental stages could be expressed as days before flowering to examine stage-specific effects of heat treatment.

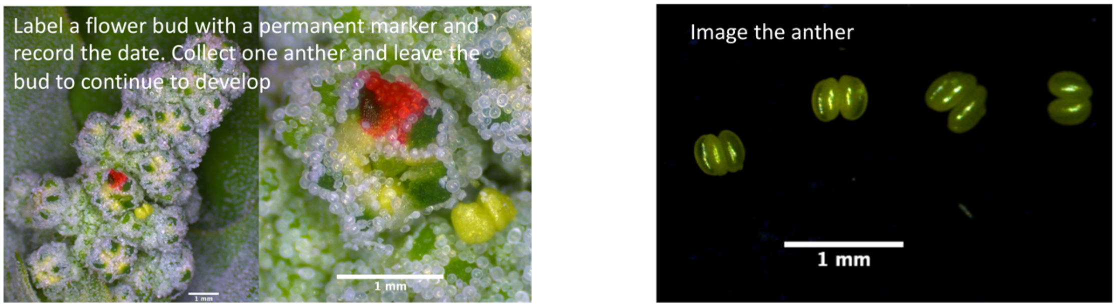

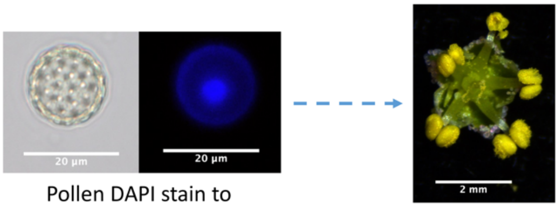

Color	Label date	Open date	Anther size	Stage by microscopy	Stage (days before flowering)
Black	10/03/2024	19/03/2024	0.315	1	-9
Orange	10/03/2024	18/03/2024	0.25	1	-8
Red	10/03/2024	19/03/2024	0.225	1	-9

Fig S2. **Schematic illustration for coordination between days before flowering and developmental stage determined by microscopy.** Young flower buds before flowering were labeled with different colors and the labelling dates were recorded. For each flower bud, a single anther was isolated and the remaining anthers were untouched, allowing them to develop for the purpose to record anthesis date. The difference between the date of labelling and the anthesis was designated as “days before flowering”. Meanwhile, the size of single isolated anthers was determined, and the pollen developmental stage was examined by DAPI staining and observed under fluorescent light microscope.

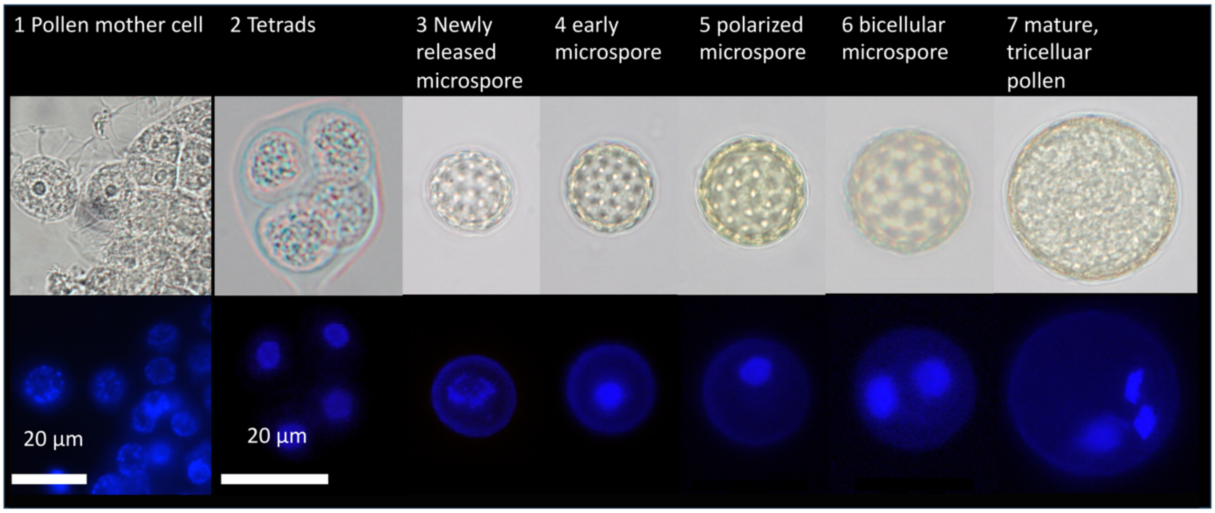

Fig S3. **Seven consecutive developmental stages identified during pollen development.** These stages were pollen mother cell, tetrads, just released microspore, early microspore, polarized microspore, bicelluar microsproe and mature pollen. Meanwhile, they were coded as stage 1-7. The pollen stage was determined by DAPI staining and observed under fluorescent light microscope.

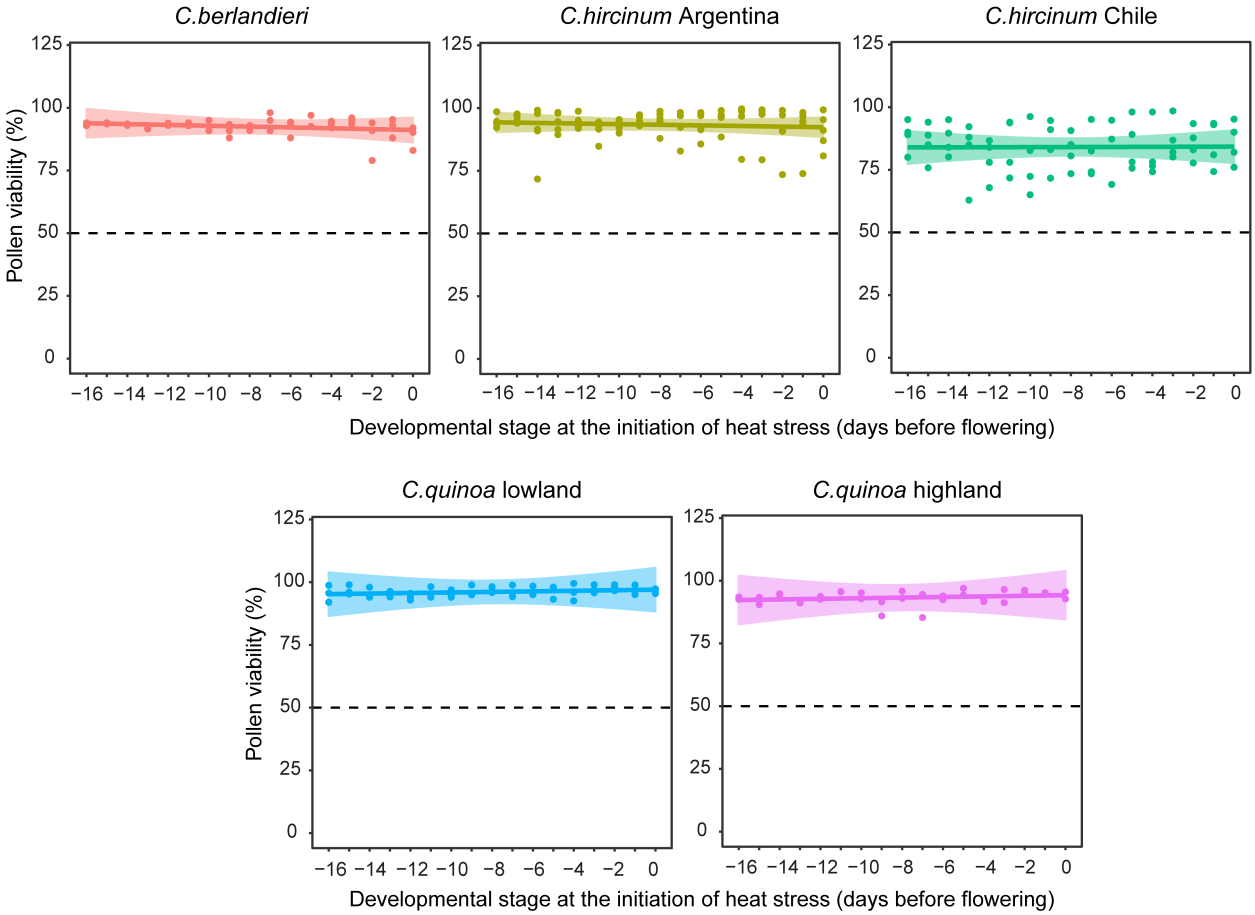

Fig S4. **Similar pollen viability of cultivated quinoa and wild relatives under control treatment**. Pollen viability was analyzed with 5 representative accessions (with one accession per group, *C.berlandieri, Chenopodium hircinum* Argentina, *Chenopodium hircinum* Chile, *Chenopodium quinoa* lowland ecotype, *Chenopodium quinoa* highland ecotype) under control temperature. After initiation of the 5-day heat treatment, data was collected from freshly open flowers on daily basis and continued for 17 days (expressed as days before flowering here in x-axis). For each day, data was collected from at least three independent replicates (individual plants) with an exception for *C.quinoa* highland. The pollen viability data was fitted with general additive models against days before flowering.

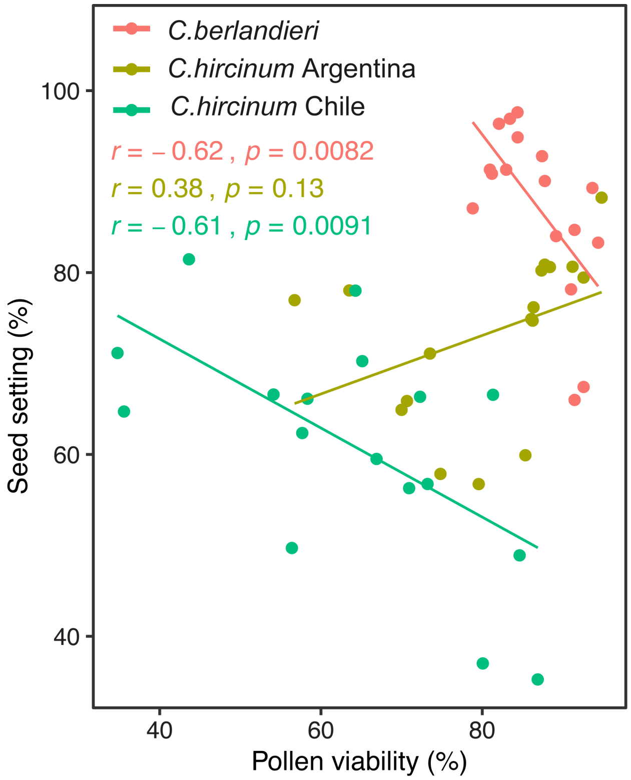

Fig S5. **Pollen viability is not a limiting factor of seed set in wild relatives.** The Pearson correlations between pollen viability and seed setting were shown for *C.berlandieri* (CB2)*,* *Chenopodium hircinum* Argentina (CHA1), *Chenopodium hircinum* Chile (CHC1) with scatter plots. The data were collected from heat treatment with precise temperature control (5 days 38/33°C, day/night). After initiation of the five- day heat treatment, pollen viability and seed setting were analyzed daily and continued for 17 days in total. Each dot in the scatter plot represents the value for each day measured and the average of at least three independent individual plant replicates.
